# Comorbidity has negligible impact on treatment and complications but influences survival in breast cancer patients

**DOI:** 10.1038/sj.bjc.6601844

**Published:** 2004-05-25

**Authors:** S Houterman, M L G Janssen-Heijnen, C D G W Verheij, W J Louwman, G Vreugdenhil, M J C van der Sangen, J W W Coebergh

**Affiliations:** 1 Eindhoven Cancer Registry, Comprehensive Cancer Centre South (IKZ), PO Box 231, 5600 AE Eindhoven, The Netherlands; 2 Department of Internal Medicine, Máxima Medical Centre, PO Box 7777, 5500 MB Veldhoven, The Netherlands; 3 Department of Radiotherapy, Catharina Hospital, PO Box 1350, 5602 ZA Eindhoven, The Netherlands; 4 Department of Public Health, Erasmus University Medical Centre, PO Box 1738, 3000 DR Rotterdam, The Netherlands

**Keywords:** comorbidity, complications, treatment, prognosis, elderly and breast cancer

## Abstract

In the present study, we investigated whether age and serious comorbid conditions influence treatment decisions, complications and survival in breast cancer patients. The Eindhoven Cancer Registry records patient, tumour and therapy characteristics of all patients diagnosed with cancer in the southern part of the Netherlands. Additional information on severity of comorbidity and serious complications was collected for a random sample of 527 breast cancer patients (aged 40 years and older). More than 70% of the patients ⩾80 exhibited high severity of comorbidity compared to 6% of those aged 40–49 years. Treatment was not influenced by severity of comorbidity. Less than 30% of the breast cancer patients had complications after diagnosis. The number of complications was not related to age or severity of comorbidity. The hazard ratio (HR) of dying for patients with low/moderate severity of comorbidity was 2.4 for those aged 40–69 years and 1.6 for those aged ⩾70 years, after adjustment for age, nodal status and treatment. For patients with high severity of comorbidity, the risk of dying was almost three times higher. Older breast cancer patients with serious comorbidity were not treated differently and did not have more complications compared to those without comorbidity, but they exhibited a worse prognosis.

Comorbidity, the coexistence of various chronic illnesses in addition to the index disease, is an increasing problem in industrialised countries due to the rising proportion of older people. In 1970, 25% of all breast cancer patients was 70 years or older and in 1999 33% (Coebergh *et al*, 2001). In a population-based study in the registration area of the Eindhoven Cancer Registry, the prevalence of comorbidity increased from 10% of patients younger than 50 to 55% of patients aged 80 years and older (Louwman *et al*, submitted). Comorbid conditions may have an effect on oncological treatment of older breast cancer patients. Several studies have shown that older patients received less extensive treatment (like adjuvant radiotherapy and chemotherapy) (Greenfield *et al*, 1987; Bergman *et al*, 1991; Yancik *et al*, 2001; Bouchardy *et al*, 2003; Louwman *et al*, submitted). Few data exist on treatment results for older breast cancer patients with serious comorbid conditions, since these patients generally are not eligible for clinical trials (Hutchins *et al*, 1999; Kemeny *et al*, 2003). Comorbidity may also have a negative impact on prognosis. Some studies have found that breast cancer patients with comorbid conditions had a lower overall survival rate compared to patients without comorbidity (Bergman *et al*, 1991; Satariano and Ragland, 1994; Yancik *et al*, 2001; Louwman *et al*, submitted).

In this study, we investigated whether age and serious comorbid conditions influenced treatment decisions, the occurrence of serious complications within 1 year after diagnosis and survival in breast cancer patients.

## MATERIALS AND METHODS

### Study population

The Eindhoven Cancer Registry (ECR) has collected data on all patients with newly diagnosed cancer in the Dutch province of North Brabant and in the northern part of the adjacent province of Limburg. The registry serves a population of about 2 million inhabitants. Information on diagnosis, staging, treatment and comorbidity (since 1993) were extracted from the medical records. The area offers good access to specialised medical care supplied in 12 general hospitals and two large radiotherapy institutes.

For this study, a random sample was taken from the database of the Eindhoven Cancer Registry (function uniform in the SAS computer package). This sample consisted of 549 women aged 40 years and older with breast cancer, diagnosed between 1995 and 1999 in eight out of 12 general hospitals in the registration area. This sample size was considered large enough for subgroup analyses according to time and costs of review of medical records. Of these 549 patients, 22 patients were excluded for the following reasons. In all, 14 clinical records could not be found in the hospitals due to migration, death or unknown reasons and eight clinical records were incomplete. These excluded patients had a mean age of 68 years (compared to 62 years for those in the study) and 59% died during follow-up (compared to 25% deaths in the study). Data of the other 527 patients were included for analyses.

### Measurements

With the approval of the treating physicians, two researchers (an epidemiologist (SH) and a surgeon (CV)) extracted additional information on severity of comorbidity, complications and performance status from the medical records. Severity of comorbidity was classified as high, moderate and low impact according to the conceptual model ‘Life Threat’ (Yancik *et al*, 1998) (Table 1

**Table 1:** Classification of severity of comorbidity, according to an adapted version of the model ‘Life Threat’ (Yancik *et al*, 1998)

**High impact**	**Moderate impact**	**Low impact**
Angina^a^	Angina^b^	Arrhythmia^b^
Arrythmia^a^	Cardiovascular diseases^b^	Alzheimer's disease^a^,^b^
Cardiac arrest^a^,^b^	Myocardial infarction^b^	Arthritis^a^
Congestive heart failure^a^,^b^	Valve disease^b^	Diabetes (medication unknown)^a^
Cardiovascular disease^a^	Anaemia^a^	Eye problems^a^
Myocardial infarction^a^	Asthma^a^	Fracture^a^
Valve disease^a^	Depression^a^	Hearing^a^
Other heart problems^a^	Deep vein thrombosis^a^	Hypertension^b^
Chronic obstructive	Gastrointestinal^a^	Liver problems^b^
Pulmonary disease^a^,^b^	Hypertension^a^	Obesity^a^
Diabetes (insulin)^a^	Lipid problems^a^	Osteoporosis^a^
Previous cancer^a^	Liver problems^a^	Parkinson's disease^a^
Renal failure^a^,^b^	Mental health^a^	Previous cancer^b^
	Stroke/TIA^a^	Stroke/TIA^b^
		Thyroid/glandular^a^
		Urinary tract (any Hx)^a^,^b^

a Under active treatment.

b No current treatment, history only.

TIA=transient ischaemic attack.

Hx=history.

). We did not include the category ‘negligible impact’ in our study, because we assume that the influence of these comorbid conditions on treatment and prognosis would have been negligible and similar to that of patients without comorbidity. For analyses of treatment, complications and survival, patients with more comorbid conditions were classified according to the most severe condition.

The functional status of the patient was extracted from the medical record, using the Karnofsky scale (Karnofsky *et al*, 1948). Five categories were distinguished according to the Eastern Co-operative Oncology Group (ECOG). For surgical patients, we also recorded the American Society of Anaesthesiologists (ASA) physical status score (House of Delegates of the American Society of Anesthesiologists, 1963). However, it proved to be impossible to include functional status and the ASA score in the analyses, because for 68% of the breast cancer patients, the functional status was not found in the medical record and for 45% the ASA score was missing.

Treatment was classified as surgery, surgery plus radiotherapy, surgery plus radiotherapy plus systemic therapy (chemotherapy and/or hormonal therapy), surgery plus systemic therapy and other therapy. Axillary nodal status was recorded on the basis of clinical and pathological examination.

Serious complications were recorded during the first year after diagnosis. Complications registered were minor infections (such as superficial wound infection), major infections (such as abscess, septicaemia), pulmonary complications (such as pneumonia), haemorrhage (requiring blood transfusion or surgery), thromboembolic complications, cardiac problems (such as cardiac insufficiency), complications due to radiotherapy (such as pneumonitis) or chemotherapy (such as severe nausea), lymphoedema and other complications.

Information on vital status of the patients was obtained from the hospital records and the death register of the Central Bureau for Genealogy. Follow-up was completed until 1 July 2003 (mean follow-up 4.7 years).

### Statistical analysis

The association between severity of comorbidity and treatment and number of complications (0, 1 and ⩾2) was analysed according to age. Differences between subgroups were tested with the *χ*^2^ test. Univariate crude 5-year survival rates were calculated according to severity of comorbidity, axillary nodal status, treatment and age. Survival time was defined as the time from diagnosis until death or the end of the study (if the patient was still alive on 1 July 2003). Differences in crude survival between categories were tested with the log-rank test. In a multivariable Cox's proportional-hazard regression analysis, independent hazard ratios (HR) for severity of comorbidity were estimated and adjusted for age at diagnosis, axillary nodal status and treatment. Survival generally decreases with age and the prevalence of comorbidity increases with age. Therefore, we estimated relative survival rates. Relative survival rates were calculated as the ratio of observed survival in the cancer patients divided by the expected survival of a group of individuals of closely similar age from the general population. The SAS computer package (version 8.2) was used for all statistical analyses (SAS Institute Inc., Cary, North Carolina, USA, 1999).

## RESULTS

For this study, we extracted a random sample of women aged 40 years and older with breast cancer from a population-based database. The distribution of age, stage and vital status in the random sample was similar to that in the whole database of the Eindhoven Cancer Registry. In total, 373 breast cancer patients were aged between 40 and 69 years and 154 patients were 70 years and older.

**Figure 1:**
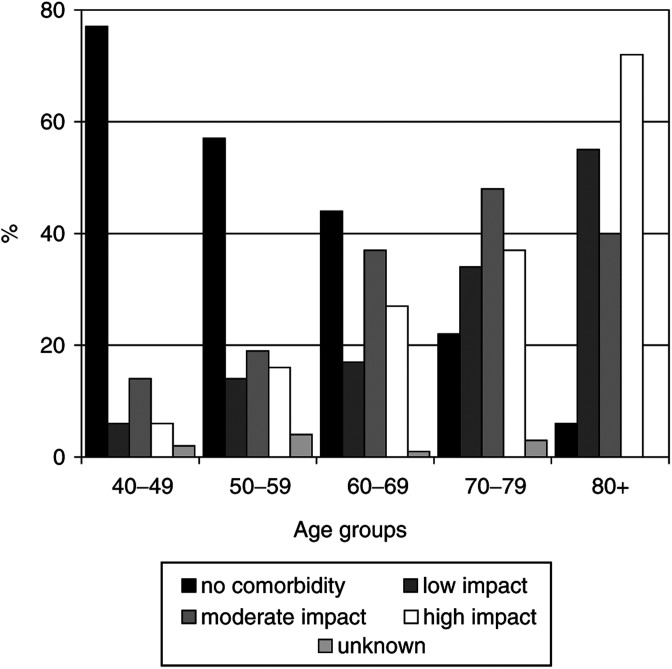
Age-specific prevalence of comorbidity among breast cancer patients diagnosed in 1995–1999 in the southern Netherlands.

In all, 77% of the patients aged 40–49 years had no concomitant disease, whereas this was only 6% of those aged 80 years and older (Figure 1

). High impact concomitant diseases were most prevalent among older patients (72% of those aged 80 years and older *vs* 6% of those aged 40–49 years).

The most common condition with high impact was hypertension under active treatment (12% of patients aged <70 years and 32% of patients aged ⩾70 years). In all, 7% of younger patients had cardiovascular diseases with high impact compared to almost 25% of older patients. Diabetes, both low and high impact, occurred more frequently in patients aged 70 years and older (10 and 8%, respectively) than in patients aged 40–69 years (2 and 3%, respectively).

**Figure 2:**
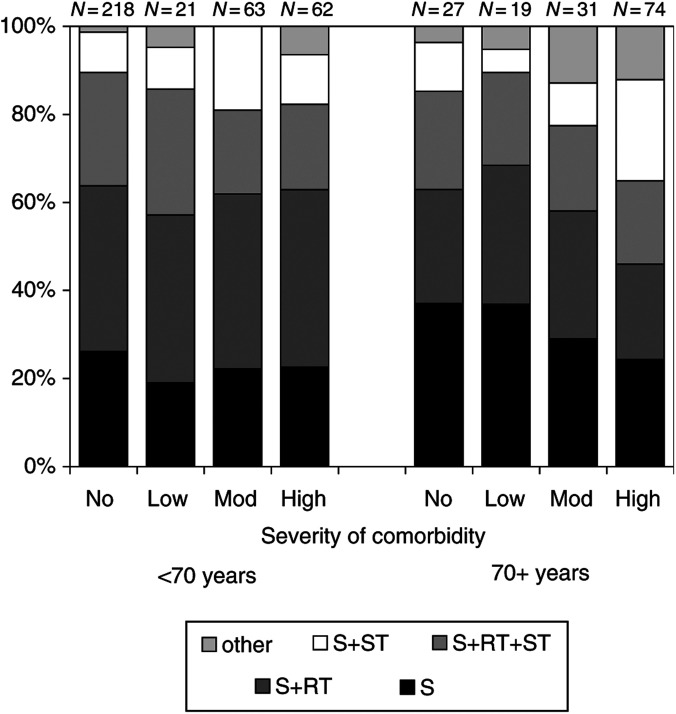
Treatment (%) of breast cancer patients diagnosed in 1995–1999 in the southern Netherlands, according to severity of comorbidity and age. S=surgery, S+RT=surgery and radiotherapy, S+RT+ST=surgery and radiotherapy and systemic therapy, S+ST=surgery and systemic therapy.

Overall, patients aged 70 years and older received significantly less radiotherapy and chemotherapy and significantly more hormonal therapy than patients under 70 years did (results not shown). The percentage of patients aged ⩾70 years receiving surgery and radiotherapy was 25% compared to 38% of those aged <70 years. Of patients aged 70 years and older, <1% received chemotherapy and 44% hormonal therapy compared to 17 and 25%, respectively, of patients younger than 70 years. For patients younger than 70, treatment was not influenced by severity of comorbidity (Figure 2

). Patients aged 70 years and older with high impact comorbidity received slightly more often surgery combined with systemic therapy, and less surgery alone or surgery in combination with radiotherapy, but this was not significant (*P*=0.7). Splitting up the variable systemic therapy in hormonal therapy and chemotherapy showed no association between type of systemic treatment and severity of comorbidity in patients aged 40–69 years (*P*=0.31) and in those aged 70 years and older (*P*=0.35) (data not shown). There was no relationship between type of surgery (breast conserving therapy or mastectomy with or without axillary dissection) and severity of comorbidity (data not shown). In all, 46% of the patients aged 40–69 years without comorbidity were treated with breast conserving therapy with axillary dissection compared to 47% of the patients with high severity of comorbidity. Of those aged 70 years and older, these percentages were 35 and 23%, respectively (*P*=0.7).

The number of patients with complications after surgery was similar for both age groups (27% of those aged younger than 70 years and 26% of those aged 70 years and older) (Table 2

**Table 2:** Age-specific prevalence of complications during the first year after diagnosis for breast cancer patients diagnosed in 1995–1999 in the southern Netherlands

	**<70 years**	**⩾70 years**
**Type of complication^a^**	**% *(N)***	**% *(N)***
No complication	71.1% (265)	69.5% (107)
Minor infection (like superficial wound infection)	11.5% (43)	9.7% (15)
Major infection (like abscess, septicaemia)	3.2% (12)	4.6% (7)
Pulmonary (like pneumonia)	0.8% (3)	1.3% (2)
Haemorrhage (requiring blood transfusion or surgery)	3.5% (13)	3.3% (5)
Thromboembolic	0.0% (0)	0.7% (1)
Cardiac (like cardiac insufficiency)	0.3% (1)	1.3% (2)
Following radiotherapy (like pneumonitis)^b^	3.5% (8)	1.4% (1)
Following systemic therapy (like severe nausea)^c^	1.5% (2)	0.0% (0)
Lymphoedema^d^	7.7% (25)	7.0% (8)
Other	3.5% (13)	2.0% (3)
Unknown	1.9% (7)	4.6% (7)

a More complications per patient possible.

b Proportion of patients who underwent radiotherapy.

c Proportion of patients who underwent chemotherapy.

d Proportion of patients who underwent axillary dissection.

). Patients aged 80 years and older had somewhat less complications (15%). Minor infection (10–12%) was the most common complication, followed by lymphoedema (7–8%). Complications following radiotherapy were more prevalent in patients younger than 70 (3%) than of those aged 70 years and older (1%). The proportion of patients aged 70 years and older with two or more complications was 0% of those without comorbidity, 6% of those with low severity, 10% of those with moderate severity and only 1% of those with high severity of comorbidity. There was no statistically significant difference in both age groups between the number of complications and the severity of comorbidity (*P*=0.6 of patients younger than 70 years and *P*=0.3 of those aged 70 years and older) (Figure 3

**Figure 3:**
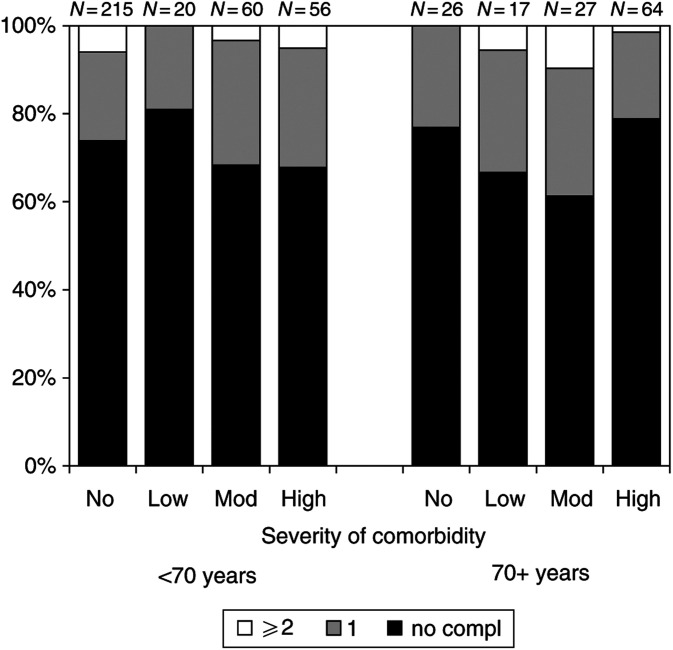
Number of complications (%) during the first year after diagnosis among breast cancer patients diagnosed in 1995–1999 in the southern Netherlands, according to severity of comorbidity and age. compl=complications.

).

The 5-year survival for patients without comorbidity was 91% for those younger than 70 years and 78% for those aged 70 years and older (Table 3

**Table 3:** Univariate and multivariable analyses of survival for breast cancer patients diagnosed in 1995–1999 in the southern Netherlands

	**<70 years**				**⩾70 years**			
	**Univariate**				**Univariate**			
**Variable**	***N* (deaths)**	**5-year survival % (s.e.)**	***P*-value**	**Multivariable HR (95% CI)^a^**	***N* (deaths)**	**5-year survival % (s.e.)**	***P*-value**	**Multivariable HR (95% CI)^a^**
*Severity of concomitant disease*			0.002				<0.001	
No comorbidity^b^	218 (24)	91 (2)		1.00	27 (6)	78 (9)		1.00
Low/moderate impact	84 (19)	84 (4)		2.43 (1.27–4.66)	50 (18)	73 (7)		1.63 (0.58–4.59)
High impact	62 (17)	80 (5)		2.87 (1.40–5.90)	74 (46)	46 (6)		2.97 (1.12–7.86)
								
*Axillary nodal status*			<0.001				0.006	
Negative^b^	194 (20)	92 (2)		1.00	67 (25)	68 (6)		1.00
Positive	123 (31)	80 (4)		2.10 (0.78–5.65)	44 (19)	62 (8)		0.69 (0.27–1.80)
Unknown/no axillary dissection	56 (10)	84 (5)		0.83 (0.27–2.55)	43 (27)	47 (8)		0.66 (0.27–1.61)
								
*Treatment*								
Surgery only^b^	91 (10)	91 (3)	0.001	1.00	44 (16)	71 (7)	0.32	1.00
Surgery and radiotherapy	142 (14)	93 (2)		0.87 (0.38–1.96)	39 (14)	67 (8)		1.27 (0.58–2.81)
Surgery and radiotherapy and systemic	87 (23)	77 (5)		1.62 (0.55–4.78)	30 (13)	70 (8)		1.35 (0.47–3.90)
Surgery and systemic	44 (8)	87 (6)		1.11 (0.33–3.74)	25 (13)	49 (11)		1.41 (0.50–4.03)
Other	9 (6)	—		d	16 (15)	—		d
								
Age (years)^c^	373 (61)	87 (2)		1.01 (0.97–1.05)	154 (71)	60 (4)		1.09 (1.03–1.16)

a Hazard ratio (HR) for death and 95% confidence interval (CI) adjusted for all other variables in the model.

b Reference category.

c As a continuous variable in the multivariable analysis.

d Not included in the multivariable model.

). Of patients aged 40–69 years, the 5-year survival rate for those with low/moderate and high impact of comorbidity was significantly lower than for patients without comorbidity (84 and 80%, respectively, *vs* 91%). The prognosis for patients aged 70 years and older was significantly worse for those with high severity of comorbidity (5-year survival 46%). In the multivariable analyses (adjusted for age at diagnosis, axillary nodal status and treatment), the risk of dying for patients younger than 70 years was 2.4 times higher for those with low to moderate comorbidity and 2.9 times higher for those with high impact comorbidity compared to patients without comorbidity. Patients aged 70 years and older with low/moderate impact had a nonsignificant higher risk of dying compared to patients without comorbidity, whereas patients with high impact comorbidity had a three times higher risk of dying. Age was also an independent prognostic factor for patients aged 70 years and older. Treatment and axillary nodal status were no significant prognostic factors after adjustment for age and severity of comorbidity. Splitting up the variable systemic therapy in hormonal therapy and chemotherapy did not alter the above-mentioned results. Using a cutoff point in the multivariable analyses of 60 years instead of 70 years still did not alter the results. The 5-year relative survival rate was not significantly lower in older patients (Table 4

**Table 4:** Relative survival rates for breast cancer patients diagnosed in 1995–1999 in the southern Netherlands

	**<70 years**		**⩾70 years**	
	**%**	**95% CI**	**%**	**95% CI**
*Severity of concomitant disease*				
No comorbidity	93	88–96	94	66–109
Low/moderate impact	87	77–94	92	72–105
High impact	83	70–92	69	51–86
				
*Axillary nodal status*				
Negative	95	90–98	87	70–100
Positive	83	74–89	86	63–103
Unknown/no axillary dissection	87	73–95	71	47–92
				
*Treatment*				
Surgery only	94	86–99	97	74–113
Surgery and radiotherapy	96	90–99	82	61–98
Surgery and radiotherapy and systemic	80	69–88	94	67–112
Surgery and systemic	90	74–98	85	48–117
Other	—	—	—	—
				
Age (years)	90	86–93	82	70–92

CI=confidence interval.

). Older women with high impact comorbidity exhibited lower relative survival rates compared to those without comorbidity, but this difference was not significant.

## DISCUSSION

This study shows that severe comorbidity at the time of cancer diagnosis negatively affected survival of breast cancer patients diagnosed in general hospitals. However, it did not appear to influence choice of treatment or the occurrence of complications after treatment.

There was no significant difference in treatment for women with breast cancer according to severity of comorbidity. This confirms the results of previous studies (Greenfield *et al*, 1987; Bergman *et al*, 1991), where age was a more important factor for choice of treatment than comorbidity. In a prospective cohort study among American breast cancer patients aged 65 years and older, no association was found between primary therapy and comorbidity (Lash *et al*, 2003). However, a study among 1196 breast cancer patients in the Kaiser Permanente Medical Care Program demonstrated that patients with two or more comorbid conditions received less adjuvant breast cancer therapy, such as radiotherapy and chemotherapy (West *et al*, 1996). In a large population-based study with all breast cancer patients diagnosed between 1995 and 1999 (*N*=6277) in the area of the Eindhoven Cancer Registry (from which our sample was derived), we found that breast cancer patients with comorbidity less frequently received adjuvant radiotherapy (50 *vs* 65%, *P*<0.0001) and less extensive surgical treatment (Louwman *et al*, submitted). An explanation for this discrepancy could be that we had too few patients in our random sample to see this effect. Another explanation could be that different classification systems for comorbidity were used.

In this population-based study that was carried out in medium-sized general hospitals in which the caseload was relatively high, we found no statistically significant difference between the number of complications and the severity of comorbidity or age. Complications following radiotherapy, however, were more prevalent in patients younger than 70 years compared to those aged 70 years and older, even after the adjustment for the higher proportion of younger patients receiving radiotherapy. Younger patients, however, did not receive a higher dose of radiotherapy. We are not aware of any other study describing the effect of comorbidity on postoperative complications in older breast cancer patients. Of patients who underwent prostatectomy, comorbidity and age did not significantly affect complications, but comorbid conditions did prolong postoperative bed-stay (Ibrahim *et al*, 1995). The latter was not recorded in this study.

Prognosis of breast cancer patients with severe comorbidity was significantly worse compared to patients without comorbidity after adjustment for age, axillary nodal status and treatment. Other studies also showed that breast cancer patients with comorbidity had a lower survival rate compared to patients without comorbidity, regardless of other prognostic factors such as age and stage of breast cancer at diagnosis (Bergman *et al*, 1991; West *et al*, 1996; Yancik *et al*, 2001; Louwman *et al*, submitted). In a longitudinal observational study carried out in the Detroit metropolitan area among women aged 40–84 years, patients with three or more comorbidities even had a 20-fold higher rate of mortality from causes other than breast cancer and a four-fold higher rate of all-cause mortality compared to patients without comorbidity (Satariano and Ragland, 1994). This means that breast cancer, and perhaps its treatment, may accelerate the course of other pathologic conditions, which might increase the risk of death from those conditions.

Owing to the relatively small number of breast cancer patients in this study, the effect of the individual comorbid conditions on prognosis could not be analysed.

Comparing the results of the above-mentioned studies, we have to take into account that the comorbidity measures used were somewhat different. In several studies (Newschaffer *et al*, 1996; West *et al*, 1996; Lash *et al*, 2003), the Charlson comorbidity method (Charlson *et al*, 1987) was used to measure comorbidity and in other studies (Greenfield *et al*, 1987; Bergman *et al*, 1991) another measure of comorbidity was used. Although the effect of severity of comorbidity according to the ‘Life Threat’ model (Yancik *et al*, 1998) has not yet been validated in breast cancer, we believe it to be the best available index to measure severity of comorbidity in which information about treatment of the comorbid condition was included. Besides this, the measure was shown to predict survival in a large population-based study of colorectal cancer patients (Yancik *et al*, 1998). Therefore, we are not surprised to find that severity of comorbidity, measured according to the ‘Life Threat’ model, was a strong prognostic factor in our study.

We extracted data from the patient's medical record as these are generally regarded as the most complete source of information on the patient's past and current health status (Satariano, 2000). However, specific information like functional status and ASA score was not consistently available across hospitals and patients. Therefore, we could not include these prognostic factors in our analyses.

Studies including older cancer patients should asses comorbidity and functional status separately, because they predict survival in older cancer patients in a different way (Extermann *et al*, 1998). The correlation between the ECOG performance status score and the Charlson comorbidity scale was only 0.14. Functional status may reflect an interactive process between cancer stage and comorbidity level, in which psychosocial factors may act as confounders. Apart from this, comorbidity and a deterioration in functional status of patients aged 70 years and older led to greater heterogeneity and therefore more individualisation of treatment protocols (Hillen and Hupperets, 2000).

We estimated crude 5-year survival rates. It is expected that the survival rate of older breast cancer patients is worse. Since the prevalence of comorbidity also increases with age, this could have biased our results. However, comorbidity retained its prognostic value after adjustment for age. The relation between comorbidity and survival was somewhat weaker but comparable if relative survival rates were calculated. Unfortunately, we had no data on cause of death of our patients, so we could not give cause-specific survival rates.

Until now, there is not enough evidence that the biological effect of treatment, like toxicity of chemotherapy and radiotherapy, is different for older breast cancer patients with comorbidity than for younger patients. Elderly breast cancer patients (aged 65 years and older) are under-represented in treatment trials. A review of 164 trials carried out by the Southwest Oncology Group showed that 49% of the total breast cancer population under investigation was aged 65 years and older and only 9% of them was included in clinical trials (Hutchins *et al*, 1999). It is therefore important that more trials are initiated that include older cancer patients in order to establish the outcome in the elderly.

In conclusion, we found that older breast cancer patients with serious comorbidity were generally not treated differently and hardly had more complications. Prognosis of breast cancer patients was influenced negatively by severe comorbidity only.
